# Supplementary material for: Two-Dimensional High-Performance Thin-Layer Chromatography with Bioautography for Distinguishing Angelicae Dahuricae Radix Varieties: Chemical Fingerprinting and Antioxidant Profiling
Source: Plants (Basel). 2024 May 13;13(10):1348. doi: 10.3390/plants13101348 (PMC11125029; doi:10.3390/plants13101348)
Supplement: Supplementary file 1 [file plants-13-01348-s001.zip › plants-2980554-supplementary.pdf]

Supplementary Material

2D-HPTLC with Bioautography for Distinguishing Angelicae Dahuricae Radix Varieties: Chemical Fingerprinting and Antioxidant Profiling

Sejin Ku [1,2,†], Geonha Park [2,†] and Young Pyo Jang [1-3,*]

[1] Department of Biomedical and Pharmaceutical Sciences, Graduate School, Kyung Hee University, Seoul 02447, Republic of Korea; zbxl0910@naver.com
[2] Division of Pharmacognosy, College of Pharmacy, Kyung Hee University, Seoul 02447, Republic of Korea; ginapark0326@khu.ac.kr
[3] Department of Integrated Drug Development and Natural Products, Graduate School, Kyung Hee University, Seoul 02447, Republic of Korea, ypjang@khu.ac.kr

* Correspondence: ypjang@khu.ac.kr; Tel.: +82-2-961-9421
† Theses authors have equally contributed; Sejin Ku and Geonha Park

Table S1. Botanical origins and identification test of Angelicae dahuricae Radix in different Pharmacopoeia

Pharmacopoeia	KP [12]	ChP [13]	JP [15]	DPRKP [16]	THP [14]
**Botanical origins**	[a]*Angelica dahurica* Bentham et Hooker f.	[a]*Angelica dahurica* (Fisch. ex Hoffim.) Benth. et Hook f.	[a]*Angelica dahurica* Bentham et Hooker filius ex Franchet et Savatier	[a]*Angelica dahurica* (Fish. et Hoffim.) Benth. et Hooker	[a]*Angelica dahurica* (Hoffm.) Benth. et Hook.f. ex Franch. et Sav.
	[b]*Angelica dahurica* Bentham et Hooker f. var. *formosana* Shan et Yuan	[b]*Angelica dahurica* (Fisch. ex Hoffim.) Benth. et Hook f. var. *formosana*			[b]*Angelica dahurica* (Hoffm.) Benth. et Hook. f. var. *formosana* Yen<br>[b]*Angelica dahurica* (Hoffm.) Benth. et Hook. f. ex Franch. et Sav. cv. 'Hangbaizhi'
**Identification test**	TLC	TLC	Fluorescence test	TLC	TLC
**Extraction**	Add 1.0 g of pulverized ADR to 20 mL of methanol, sonicate for 60 min, filter, evaporate the filtrate to dryness, add 2 mL of methanol to the residue to dissolve, and use it as the test solution	Add 0.5 g of pulverized ADR to 10 mL of ether, soak for 1 hour, shake frequently, filter, evaporate the filtrate to dryness, add 1 mL of ethyl acetate to the residue to dissolve, and use it as the test solution	Add 0.2 g of pulverized ADR to 5 mL of ethanol(95), shake for 5 min, filter, and used the filtrate as the test solution	Add 0.2 g of pulverized ADR to 5 mL of ethanol, put in a water bath for 5 min, and used the supernatant as the test solution	Add 2.0 g of pulverized ADR to 10 mL of methanol, sonicate for 30 min, filter, and use the filtrate as the test solution
**Developing solvent**	Hexane, ethyl acetate 2:1 (v/v)	Petroleum ether, ether 3:2 (v/v)	n/a	Petroleum ether, ether 3:2 (v/v)	Petroleum ether, ether 3:2 (v/v)
**Developing condition**	Application volume: 2 μL<br>Developing distance: 10 cm	Application volume: 4 μL<br>Developing distance: n/a	n/a	Application volume: 5 μL<br>Developing distance: n/a	Application volume: 4 μL<br>Developing distance: 5~10 cm
**Detection**	UV 254 nm	UV 365 nm	UV 365 nm	UV 365 nm	UV 365 nm
**Reference compound**	n/a	Isoimperatorin<br>(1 mg/mL in ethyl acetate)	n/a	Byakangelicin<br>(1 mg/mL in ethanol)	Isoimperatorin, imperatorin<br>(1 mg/mL in ethyl acetate)
**Criteria**	Spots on Rf 0.45 and 0.7	Fluorescent spot appears corresponding to the reference compound	A blue to blue-purple fluorescence develops	Fluorescent spot appears corresponding to the reference compound	Fluorescent spot appears corresponding to the reference compounds

[a]*Angelica dahurica* (Hoffm.) Benth. & Hook.f. ex Franch. & Sav. **(AD);** [b]*Angelica dahurica* var. *formosana* (H.Boissieu) Yen **(ADF)**
KP: the Korean Pharmacopoeia; ChP: the Pharmacpoeia of the People's Republic of China; JP: the Japanese Pharmacopoeia; DPRKP: the Pharmacopoeia of Democratic People's Republic of Korea; THP: the Taiwan Herbal Pharmacopoeia

Table S2. Detailed information of the collected samples

No.	Name	Origin	Purchased	Expiration date	GMP
01	Angelicae Dahuricae Radix	Korea (Bonghwa, Gyeongsangbuk-do)	Chamchowon	Apr. 27th, 2025	O
02	Angelicae Dahuricae Radix	Korea	Dongyang Herb	Mar. 1st, 2024	O
03	Angelicae Dahuricae Radix	Korea (Andong, Gyeongsangbok-do)	On Herb	Apr. 11st, 2024	O
04	Angelicae Dahuricae Radix	Korea (Yeongyang, Gyeongsangbok-do)	Gwangmyeongdang Pharmaceutical	Apr. 27th, 2025	O
05	Angelicae Dahuricae Radix	Korea	World Herb	Jan. 1st, 2024	O
06	Angelicae Dahuricae Radix	Korea (Yeongyang, Gyeongsangbok-do)	Nature World	Nov. 15th, 2024	O
07	Angelicae Dahuricae Radix	Korea (Bonghwa, Gyeongsangbok-do)	Human Herb	Jul. 5th, 2025	O
08	Angelicae Dahuricae Radix	Korea (Yeongyang, Gyeongsangbok-do)	Hyungryul Pharmaceutical	Nov. 30th, 2024	O
09	Angelicae Dahuricae Radix	Korea (Jecheon, Chungcheongbuk-do)	Sunil Pharmaceutical	Mar. 27th, 2025	O
10	Angelicae Dahuricae Radix	Korea	Dongui Herb	Jan. 1st, 2025	X
11	Angelicae Dahuricae Radix	China	Chamchowon	Nov. 23th, 2024	O
12	Angelicae Dahuricae Radix	China	Dongyang Herb	Nov. 14th, 2025	O
13	Angelicae Dahuricae Radix	China	On Herb	Jan. 19th, 2024	O
14	Angelicae Dahuricae Radix	China	Gwangmyeongdang Pharmaceutical	Jul. 18th, 2025	O
15	Angelicae Dahuricae Radix	China	World Herb	Jun. 21st, 2025	O
16	Angelicae Dahuricae Radix	China	Nature World	Jan. 3rd, 2024	O
17	Angelicae Dahuricae Radix	China	Human Herb	Apr. 27th, 2025	O
18	Angelicae Dahuricae Radix	China	Hyungryul Pharmaceutical	Oct. 3rd, 2024	O
19	Angelicae Dahuricae Radix	China	Gwangdeok Pharmaceutical	Jan. 19th, 2025	O
20	Angelicae Dahuricae Radix	China	Hyunjin Pharmaceutical	Apr. 21st, 2024	O

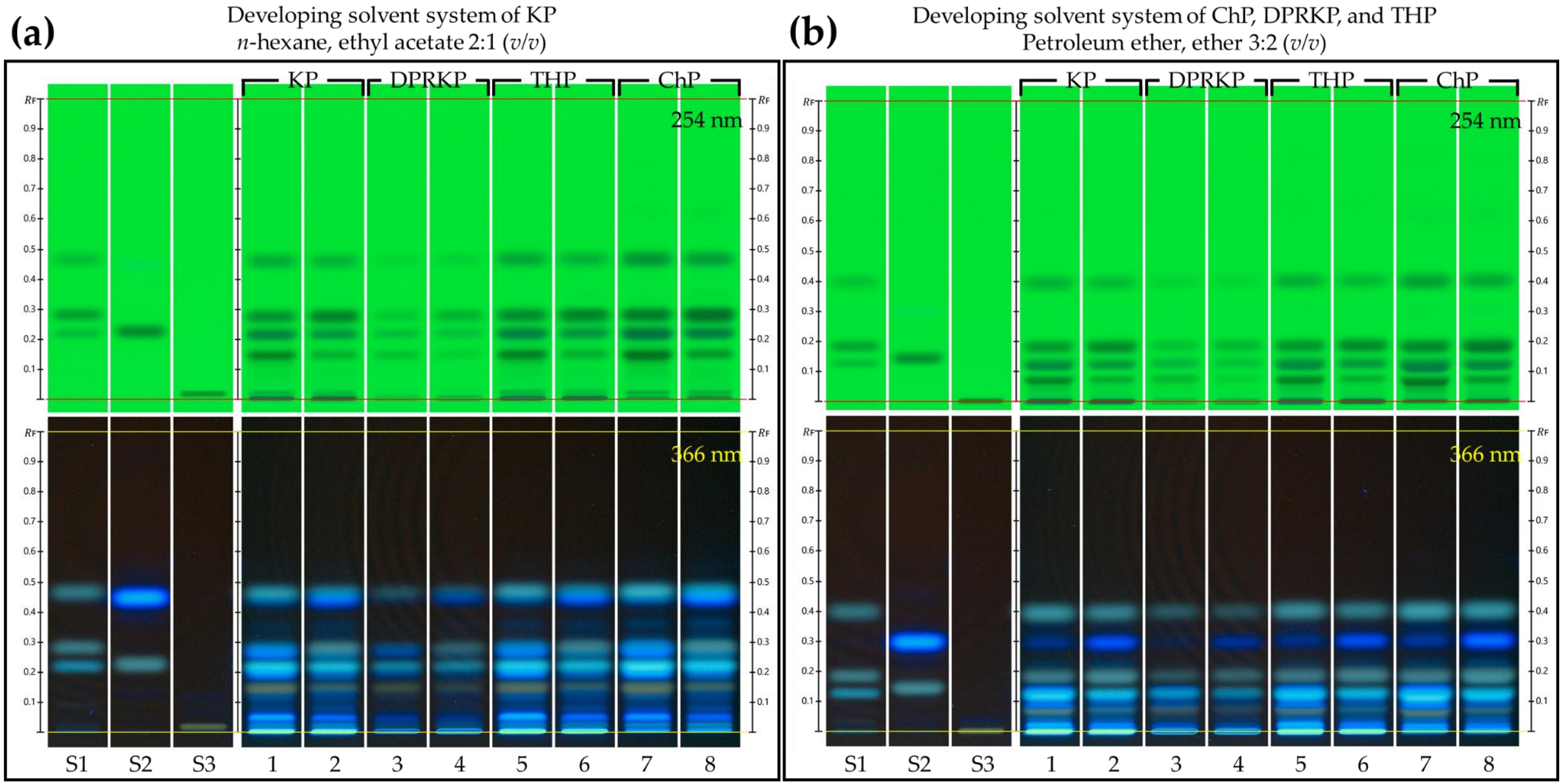

Figure S1. The HPTLC chromatograms of different pharmacopoeias conditions: **(a)** with developing solvent system of *n*-hexane and ethyl acetate (2:1, *v*/*v*) and **(b)** with developing solvent system of petroleum ether and ether (3:2, *v*/*v*). S1: oxypeucedanin, imperatorin, and isoimpertorin (as R_F increasing); S2: xanthotoxin and suberosin (as R_F increasing); S3: byakangelicin; 1: KP extract of AD [12]; 2: KP extract of ADF [12]; 3: DPRKP extract of AD [16]; 4: DPRKP extract of ADF [16]; 5: THP extract of AD [14]; 6: THP extract of ADF [14]; 7: ChP extract of AD [13]; 8: ChP extract of ADF [13]
